# New Parameter for In-Office Dental Bleaching

**DOI:** 10.1155/2016/6034757

**Published:** 2016-06-07

**Authors:** Cristina Dupim Presoto, Janaina Freitas Bortolatto, Priscila Petrucelli Freire de Carvalho, Tamara Carolina Trevisan, Michael Christopher Floros, Osmir Batista de Oliveira Junior

**Affiliations:** ^1^Department of Restorative Dentistry, Araraquara Dental School, Universidade Estadual Paulista (UNESP), 1680 Humaitá Street, 14801-903 Araraquara, SP, Brazil; ^2^Departments of Physics & Astronomy and Chemistry, Trent Centre for Biomaterials Research, Trent University, Peterborough, ON, Canada K9J 7B8

## Abstract

Dental bleaching is considered a conservative and biologically safe treatment for discolored teeth. Despite this, one of the major undesirable effects of bleaching is dentin sensitivity which may occur during and after treatment. To address these sensitivity issues, new dental bleaching preparations with lower concentrations of hydrogen peroxide (H_2_O_2_) have recently been introduced to the market. This paper presents a clinical case report of a 20-year-old female patient admitted to the Araraquara Dental School, UNESP, Brazil. The patient underwent dental bleaching using one of the new products with reduced hydrogen peroxide concentration, Lase Peroxide Lite 6%, a 6% H_2_O_2_ gel containing titanium oxide nanoparticles doped with nitrogen (6% H_2_O_2_/N-doped TiO_2_).

## 1. Introduction

The demand for aesthetic dentistry has increased continuously [1–4], and the smile's appearance has become an important part of the social attractiveness of a person and their interactive communication skill [1, 2, 4, 5].

Currently, dental bleaching has been recognized as an effective method for the treatment of discolored teeth, being considered a conservative and biologically safe type of treatment [4, 6–9].

Hydrogen peroxide (H_2_O_2_) is a chemical substance with high oxidative power and is the most widely used agent for in-office teeth whitening at concentrations ranging mainly from 25 to 35% [10–13]. Peroxide agents are highly unstable and when in contact with the tissue, they dissociate into water, oxygen, and free radicals, the latter accounting for the observed bleaching effect due to their ability to oxidize organic pigments [9]. It is known that the diffusion of H_2_O_2_ through the dentin depends on the concentration of the gel, the period of time that the agent is in contact with the tooth [13], and the thickness of the dental structure [8, 14].

One of the major undesirable effects of bleaching is tooth sensitivity [4, 7, 9, 12, 13, 15–18] that occurs during and after the treatment and may represent a degree of biological damage to the dentin-pulp complex [4, 7, 9, 13, 15–17]. There are many factors that are known to increase sensitivity, such as high concentrations of H_2_O_2_, high enamel permeability, prolonged use of bleaching agents, heat during application through accelerator lamps, and differences in the structural morphology of enamel and dentin with pores which facilitate the infiltration of bleaching [8, 12, 15, 16]. Sensitivity issues have led some manufacturers to release bleaching gels with lower concentrations of H_2_O_2_ in order to minimize the side effects produced by peroxide radicals [9, 13, 17].

Aiming to increase both efficacy and safety, new bleaching agents with lower concentrations of H_2_O_2_ based on the catalytic action of a nanoparticle semiconductor additive such as titanium dioxide (TiO_2_) activated by light sources [9, 16–20] are now entering the market [4, 9, 13, 16–20]. A significant reduction in the sensitivity of bleaching treatments which use nitrogen doped titanium dioxide and 15% H_2_O_2_ concentration has been reported in the literature [8, 9, 16–18]. Nevertheless, bleaching agents with 6% H_2_O_2_/N-doped TiO_2_ are innovative and significantly lower in peroxide concentration and, to our knowledge, there are few clinical studies evaluating in-office tooth bleaching with agents at this concentration [17, 18]. Thus, this study aims to present a case report using this new product.

## 2. Case Presentation

A female patient, PPFC, 20 years old, attended the Operative Dentistry Clinic at Araraquara Dental School, UNESP, Brazil, and reported dissatisfaction with the aesthetics of her anterior teeth. Despite the midline diastema between central incisors, the factor that most bothered her was the color of her teeth. During the clinical exam, there were no factors observed which would contraindicate the use of tooth bleaching. As the patient was young, we decided to use a bleaching agent with a minimal H_2_O_2_ concentration. We used a new product, Lase Peroxide Lite 6% (6% H_2_O_2_/N-doped TiO_2_) (DMC Equipment, São Carlos, SP, Brazil), which is activated by a light source. The light source used was a Whitening Lase II (DMC Equipment, São Carlos, SP, Brazil), a new type of in-office photocatalytic equipment composed of six violet LEDs (405 nm wavelength) and three infrared lasers (808 nm).

**Figure 1:**
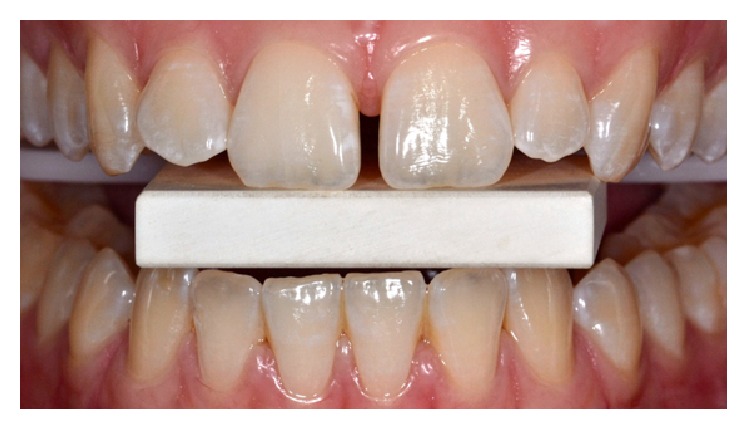
Initial picture taken with calibration block.

**Figure 2:**
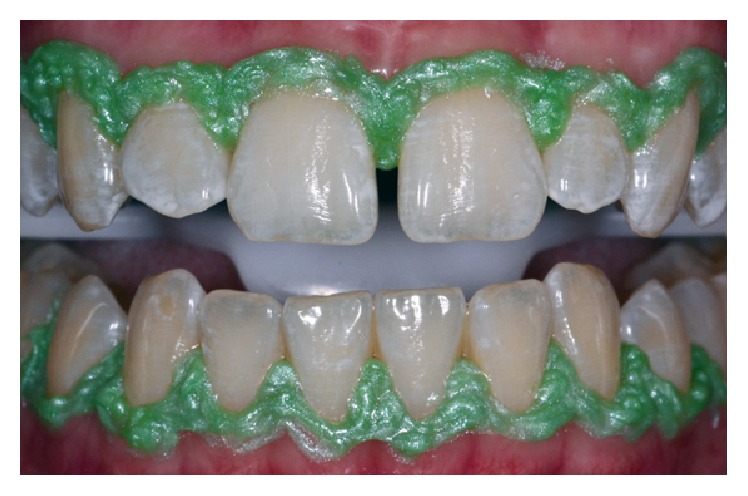
Gingival barrier properly applied and light-cured.

Before beginning the treatment, initial photography with a ceramic block for calibration was performed and retained for subsequent analysis using ScanWhite, a specific software program for the objective assessment of tooth bleaching levels based on computational processing of digital images (Figure 1). Subsequently, professional dental prophylaxis was performed with a Robinson brush with a pumice and water paste. A gingival barrier (Lase Protect, DMC, São Carlos, SP, Brazil) was then applied and light-cured for 10 seconds for each dental element (Figure 2).

**Figure 3:**
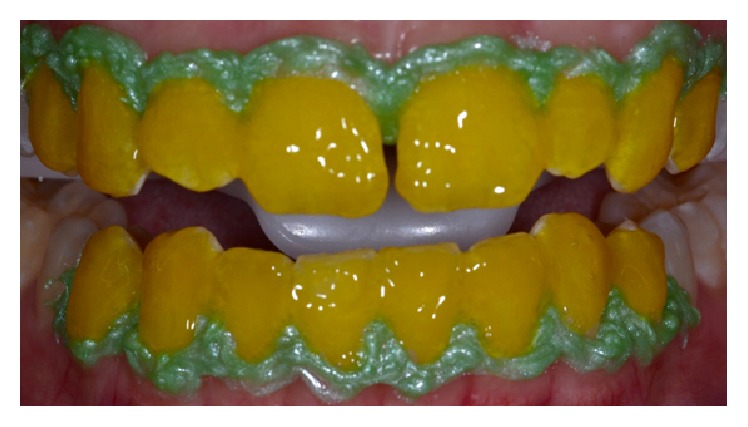
Bleaching gel applied to the buccal surface of the upper and lower teeth.

The bleaching agent was mixed and applied in homogeneous layers on the buccal surfaces of the upper and lower anterior teeth (Figure 3). The 6% H_2_O_2_/N-doped TiO_2_ gel is provided in two phases, a peroxide and a thickener, which should be mixed in proportions of 3 drops of peroxide for 1 drop of thickener. In this case, for whitening the upper and lower arches until the premolars region, we used a total of 18 drops of peroxide and 6 drops of thickener. After the gel's application, it was kept in contact with dental surface for 12 minutes and photocatalyzed by LED/laser light, alternating the irradiation between the upper and lower arches every minute. Afterwards, the bleaching gel was removed and a new application sequence was performed. Three clinical sessions were held with two application cycles of the bleaching agent in each of them and a 7-day interval between the sessions.

**Figure 4:**
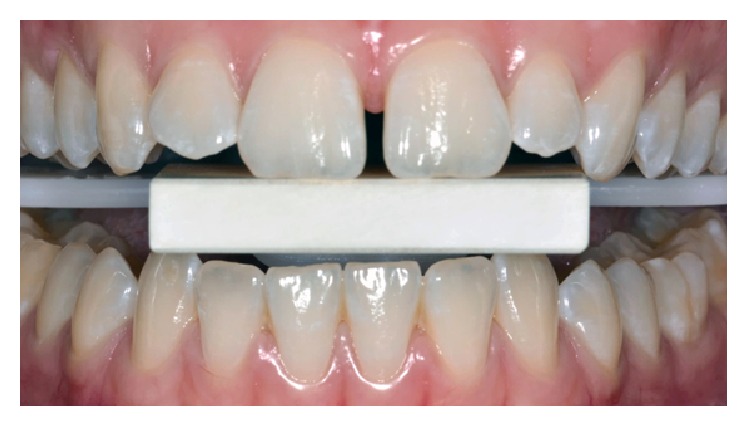
Final picture taken with calibration block.

**Figure 5:**
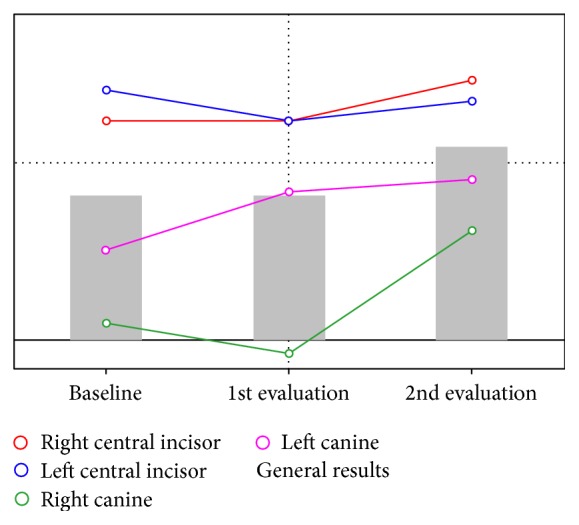
Overall result of bleaching.

One week following the end of the bleaching treatment, a new photography session was performed with the same calibration block (Figure 4). The initial and final images were compared in ScanWhite to determine how much the teeth responded to the whitening procedure. The images were first transmitted to the software and a calibration was performed. Next, for each picture, an area was selected in the middle third of labial surface of the anterior teeth. The result showed 22 tones of whitening for the upper right central incisor and 50 tones for the right canine (Figure 5). The patient reported that she was very satisfied with the final color of her teeth.

Tooth sensitivity was measured by a visual analogue scale (VAS), which provides a range of scores from 0 to 100, where 0 represents no pain and 100 represents extreme pain. At the end of each clinic session, the patient was asked to mark on the scale their perceived sensitivity both during and after the session. She reported sensitivity only during the first application in the second session and this was classified as low, with an intensity of 20% in a shock sensation and with a 1-second duration.

## 3. Discussion

The presence of sensitivity to bleaching treatments with high concentration gels and the search for an alternative with both efficacy and safety were the main reasons why bleaching agents with lower concentrations of H_2_O_2_ were introduced to the market [4, 8, 13, 17, 18]. Initially, it was believed that a high concentration gel and long contact time with dental structure would be required to obtain greater efficacy with bleaching procedures [9, 21]. Nonetheless, the use of 15% H_2_O_2_/TiO_2_ photocatalyzed by LED was recently reported and demonstrated that, besides having lower sensitivity compared to products with 35% concentration, this preparation also provided greater bleaching efficacy [8, 9, 16, 17, 20]. We also observed this finding in the clinical case presented here using a preparation with only 6% H_2_O_2_.

The presence of N-doped TiO_2_ semiconductor allows a reduction in the required concentration of H_2_O_2_, which improves the biocompatibility of the final product by significantly reducing dental sensitivity during and after the procedure, directly increasing the safety of the whitening procedure [9, 17–19]. By incorporating N-doped TiO_2_ nanoparticles, it is necessary to photocatalyze the gel with a light source in order to improve its action. This new class of bleaching agents is safer and effective for promoting bleaching with a reduced concentration of free radical peroxides, thereby minimizing damage to the tooth structure [22].

In a clinical study using a 6% H_2_O_2_ varnish system for in-office whitening, da Mata and Marques [10] reported that none of the patients showed any sensitivity, which was a common side effect upon using gels with high peroxide concentrations. By using a 6% H_2_O_2_ varnish, Calatayud et al. [23] also demonstrated that the application of the gel showed significant clinical efficacy when applied to in-office bleaching and also when applied by patients themselves at home.

In the clinical case described in this paper, upon comparing the results before and after the bleaching procedure, the choice for central incisors and canines teeth was done to ensure a more homogeneous sample, as central and lateral incisors usually have the same color and, together, could distort the results of the hemiarch's mean score [23–25]. In Figure 5, a major color alteration is observed for the canines when compared to central incisors. The results showed that the use of these new bleaching gels provides color alteration needed for patient satisfaction in addition to lowering dental sensitivity. Therefore, this case reported a successful bleaching treatment for young patients, combining the great aesthetic results, as desired by patients, and also improved biocompatibility and safety from a lower concentration of bleaching gel.
